# Exploring Generation Z and Young Millennials’ Perspectives of a Spiritual Self-Care App and Their Spiritual Identity (Skylight): Qualitative Semistructured Interview Study

**DOI:** 10.2196/54284

**Published:** 2023-12-28

**Authors:** Susanna Y Park, Jacqlyn Yourell, Kelsey L McAlister, Jennifer Huberty

**Affiliations:** 1 Skylight Radiant Foundation Salt Lake City, UT United States; 2 Fit Minded Inc Phoenix, AZ United States

**Keywords:** Gen Z, Generation Z, millennial, GenZennials, millennials, young adult, young adults, spirituality, spiritual, self-care, mental health, sleep, mobile health, app, apps, digital health, experience, experiences, attitude, attitudes, opinion, perception, perceptions, perspective, perspectives, acceptance, interview, interviews, thematic analysis, mobile phone

## Abstract

**Background:**

Generation Z and young millennials (ages 18-35 years), collectively referred to as GenZennials, are connected to technology and the internet like no other generation before them. This has mental health implications, such as increased rates of anxiety and stress. Recent research has shown that app-based mental health interventions can be useful to address such mental health concerns. However, spirituality is an untapped resource, especially since GenZennials largely identify as spiritual and already integrate spiritual practices into their self-care.

**Objective:**

There were four objectives to this study: (1) comprehensively explore reasons why GenZennials use a spiritual self-care app (ie, Skylight; Radiant Foundation), (2) understand how GenZennials identify spiritually, (3) understand the app’s relevance to GenZennials, and (4) gather feedback and suggestions to improve the app.

**Methods:**

Semistructured interviews were conducted with 23 GenZennials (ages 18-35 years; mean 28.7, SD 5.0 years; n=20, 87% female) who used the Skylight app. Interviews were 30 to 60 minutes and conducted on Zoom. Thematic analysis was used to analyze interviews.

**Results:**

Five major categories emerged from the analysis, each encompassing one to several themes: (1) reasons for using the Skylight app, (2) content favorites, (3) defining spiritual identity, (4) relevance to GenZennials, and (5) overall improvement recommendations. Participants used the app for various reasons including to relax, escape, or ground themselves; improve mood; and enhance overall health and wellness. Participants also cited the app’s variety of content offerings and its free accessibility as their primary reasons for using it. Most participants identified themselves as solely spiritual (8/23/35%) among the options provided (ie, spiritual or religious or both), and they appreciated the app’s inclusive content. Participants felt that the app was relevant to their generation as it offered modern content (eg, spiritual self-care activities and short content). Participants recommended adding more personalization capabilities, content, and representation to the app.

**Conclusions:**

This is the first study to qualitatively explore GenZennials’ perspectives and the use of a spiritual self-care app. Our findings should inform the future creation and improvement of spiritual self-care apps aimed at cultivating GenZennials’ spiritual and mental well-being. Future research is warranted to examine the effects of using a spiritual self-care app on GenZennial mental health.

## Introduction

Generation Z (Gen Z; those born between 1995 and 2012) and young millennials (those born between 1981 and 1996) [1], collectively referred to as GenZennials, are a unique population as they came of age in the digital age. This time period is characterized by the ability to easily exchange information and quickly access vast amounts of data through the internet. GenZennials conduct much of their lives in the digital world, using digital platforms for communication, education, finances, and more. Over 90% of GenZennials have access to smartphones [2], and they report spending more time on their digital devices than previous generations [3]. This constant connectivity contributes to a sense of being “always on” [4]. The inability to disconnect carries several mental and physical health consequences, highlighting the need for resources to support GenZennials’ mental health.

GenZennials in the United States report higher rates of mental health problems compared to other generations [5]. According to the 2021 McKinsey Consumer Health Insights Survey that included 2906 US respondents, Gen Z had the least positive outlook on life, the most emotional distress, and more diagnoses of conditions such as substance abuse and mental illness relative to older generations [6]. Over half of the US Gen Z (57%) and nearly half of the US millennials (46%) say they have experienced anxiety and depression symptoms [7]. In a survey of over 23,000 people, nearly half (48%) of Gen Z and 38% of millennials reported being stressed or anxious all or most of the time [8]. There is a clear and urgent need to address the unique mental health needs of GenZennials in a way that resonates with them and is tailored to their experiences.

Leveraging spirituality is one potential solution to address GenZennials’ unique mental health needs. Spirituality is a broad concept in which individuals seek connection to themselves, to others, to nature, and to something greater than themselves [9]. Most Gen Z (77%) consider themselves spiritual [10], and over half (51%) of millennials report feeling deep spirituality at least weekly [11]. GenZennials define spirituality as “faith unbundled” [10], fluidly combining religious and nonreligious elements such as gratitude, fasting, prayer, art, affirmations, time in nature, and alternative practices (ie, tarot) [10,12,13]. Given the link between spirituality and mental health and GenZennials’ interest in spiritual practices [11,14], there is a need to understand how GenZennials view spirituality and how this may help them manage their mental health.

Research shows that there is a positive association between spirituality and mental health outcomes [15,16]. A review summarizing the associations of spirituality and mental health found that higher levels of spirituality were linked to fewer symptoms of depression, lower suicidality, and less substance abuse [16]. Additionally, a systematic review of randomized control trials suggests that spirituality interventions may improve outcomes such as anxiety, depression, stress, and alcoholism in adults [17]. Compared to those with low spirituality, highly spiritual adults report greater quality of life [18]. A recent cross-sectional study on 475 US GenZennial users of a spiritual self-care app found that more frequent app use was significantly associated with lower self-reported anxiety [19]. Thus, spiritual self-care apps may be a promising way to address poor mental health in GenZennials, and additional efficacy research is needed.

Skylight (Radiant Foundation) is a free spiritual self-care app developed to cultivate personal spiritual well-being and improve well-being. To our knowledge, Skylight is one of the few spiritual well-being apps available that is nondenominational and aims to increase spiritual self-care access. In a recent cross-sectional survey [19], GenZennial Skylight users (N=475) reported downloading the Skylight app to improve spiritual well-being (n=130, 27.4%) and overall health (n=125, 26.3%). Research is needed on app-based spiritual practices as a potential mental health solution for GenZennials.

The purpose of this paper is to provide a more nuanced understanding of how users engage with and perceive a spiritual self-care app. The aims of this study are to (1) understand why GenZennials use a spiritual self-care app, Skylight; (2) explore how GenZennials identify spiritually; (3) determine Skylight’s relevance to GenZennials; and (4) gather feedback to improve the app. Findings from this study may inform the product and content of spiritual self-care apps for GenZennials. Additionally, results will guide the design of a randomized controlled trial examining the effects of using a spiritual self-care app on spiritual well-being and mental health outcomes in this population.

## Methods

### Study Design

This study used an exploratory qualitative approach using semistructured interviews.

### Ethical Considerations

This study was approved by Solutions Institutional Review Board (protocol 2023-Nov-0097) as part of a larger cross-sectional study [19]. All participants in this study provided electronic consent prior to participating in the interview. Participant confidentiality was maintained by deidentifying any personal information prior to analysis. Participants were able to cancel the interview and withdraw from the study at any time.

### Recruitment and Participants

Participants were recruited from June 2023 to September 2023 through 2 methods. First, at the end of a prior cross-sectional survey on Skylight [19], GenZennial (aged 18 to 35 years) respondents were asked about participating in an interview. Second, a recruitment banner in the Skylight app asked users if they were interested in an interview. For both recruitment methods, interested individuals scheduled interviews via a Calendly link (a web-based scheduling software; Calendly) and email. Eligibility criteria required participants to be 18 to 35 years of age at the time of the interview, a US resident, and a current Skylight user. Ineligible individuals were those who had never used the mobile or web version of Skylight. Participants confirmed their eligibility in Calendly before confirming their interview time and were sent electronic consent forms via DocuSign (DocuSign, Inc). The interviewer confirmed with participants via email that they were 18 to 35 years of age. If participants were deemed ineligible after scheduling an interview, the interviewer canceled the interview and notified them via email. Participants received US $25 Amazon gift cards as compensation.

### The Skylight App

Information about Skylight has been reported elsewhere [19]. Briefly, Skylight is a free spiritual self-care app, supported by the Radiant Foundation, to cultivate an inclusive space for faith and spirituality. The app features various practices, including prayer, yoga, meditation, and affirmations. We chose the Skylight app for this study because it is the only app to our knowledge that implements spiritual self-care completely free of cost.

### Data Collection

From June 2023 to September 2023, a total of 23 participants completed semistructured interviews conducted by 3 researchers (SYP, JY, and KLM) trained in qualitative methods. The interviewers had no prior relationships with the interview participants. At the start of each interview, interviewers briefly reiterated the purpose of the study and reviewed the informed consent form, including permission for audio recording. Interviewers reminded participants that any identifiable data would be removed to protect their confidentiality. Participants were encouraged to share their honest thoughts about the app and were told that they had the right to terminate the interview at any time.

All interviews were conducted on Zoom (Zoom Video Communications), lasting 30 to 60 minutes each. SYP created a semistructured interview guide (Table 1) to maintain consistency across all interviews. The guide drew from a prior cross-sectional survey [19] that quantitatively examined Skylight user engagement (ie, frequency, content used, and use reason), perceptions of the app (ie, app purpose or scope), and views on spiritual self-care (ie, definition and importance). To explore these topics in greater depth, researchers asked participants about their reasons for using Skylight, how they identify spiritually, Skylight’s generational relevance, and suggestions for improvement. Demographic information was collected at the end of the interview. Interviews were audio recorded with participant consent and transcribed using Otter.ai (Otter.ai Inc). For each interview, researchers also took notes directly in the interview guide. All audio files and notes were transcribed verbatim, deidentified, and saved using unique participant IDs.

**Table 1:** Semistructured interview guide.

Questions	Probes
What prompts you to use Skylight? or What are you using Skylight for?	Are you using it daily or are you using it when you need it? If you use it when you need it—what are you using it for?
Do you consider yourself spiritual and/or religious?	If yes, what role does Skylight have in your spiritual practice? If no, what role does Skylight have in your life in general?
How does Skylight pertain/relevant to your generation?	In what ways do you think it targets your generation? How do you think it could better target your generation?
What would you change about Skylight?	If you could create your own content, what would that look like? What do you want to see on the app that is not there right now?
Do you have further feedback that you would like to provide or is there anything I have not addressed that you would like to speak on?	Gather demographics and end interview

### Analysis

To ensure rigor, researchers (SYP and JY) concurrently collected and analyzed data using recommended qualitative methods [20,21]. The researchers conducted a thematic analysis in Dedoose (version 9.0.107; Socio Cultural Research Consultants, LLC) [22], which involves identifying common themes in qualitative data [23]. A codebook with a priori themes was developed based on the interview questions. SYP and JY independently coded 2 transcripts and updated the codebook with additional themes and subsequent codes that emerged. Themes and codes were added when appropriate throughout data analysis, and all transcripts were reviewed with the finalized codebook to ensure all themes and codes were captured. Thematic saturation was established by iteratively reviewing codes during the analysis to confirm that no new codes emerged. SYP and JY calculated Cohen κ, which measures interrater reliability, to determine sufficient agreement (κ=0.67 for transcript 1 and κ=0.82 for transcript 2). κ scores exceeded the 0.61 threshold for acceptable agreement [24]; data may be coded independently after obtaining acceptable agreement [25]. After reviewing discrepancies, the researchers divided and independently coded the remaining transcripts. After all the transcripts were coded, SYP and JY reviewed the final codes across all transcripts and agreed on the final themes.

## Results

### Participant Characteristics

In total, 23 Skylight users completed semistructured interviews. Participants were 18 to 35 years of age (mean 28.7, SD 5.0 years) and predominantly female (n=20, 87%). More than half of the participants were White (n=13, 57), followed by those who identified as multiracial (n=5, 22%), Black (n=3, 13%), and Middle Eastern or North African (n=2, 9%). Some (n=7, 30%) participants indicated they were not affiliated with a religion. Additional demographic characteristics can be found in Table 2.

**Table 2:** Demographic characteristics of Skylight users (N=23).

Demographics			Values
Age (years), mean (SD)			28.7 (5.0)
**Sex, n (%)**			
	Male	2 (9)	
	Female	20 (87)	
	Nonbinary	1 (4)	
**Sexual orientation, n (%)**			
	Straight (heterosexual)	18 (78)	
	Gay or lesbian	1 (4)	
	Bisexual	1 (4)	
	Pansexual	1 (4)	
	Queer	1 (4)	
	Prefer not to say	1 (4)	
**Race, n (%)**			
	Arab or non-Arab North African or Middle Eastern	2 (9)	
	Black, African American, or Native African	3 (13)	
	Hispanx or Latinx	3 (13)	
	White, European American, or Caucasian	13 (57)	
	Multiracial	5 (22)	
**Annual household income (US $), n (%)**			
	Less than 50,000	15 (65)	
	50,000-74,999	3 (13)	
	75,000-99,999	1 (4)	
	150,000-199,999	1 (4)	
	200,000 or more	1 (4)	
	Prefer not to say	2 (9)	
**Education level, n (%)**			
	High school	5 (22)	
	Some college	9 (39)	
	Associate degree	2 (9)	
	Bachelor degree	6 (26)	
	Graduate degree	1 (4)	
**Employment status, n (%)**			
	Employed	11 (48)	
	Unemployed	4 (17)	
	Homemaker or stay-at-home caregiver	2 (9)	
	Student	6 (26)	
**Relationship status, n (%)**			
	Single	7 (30)	
	In a relationship	11 (48)	
	Married	3 (13)	
	Divorced or separated	2 (9)	
**Present religion, if any, n (%)**			
	Agnostic	1 (4)	
	Catholic	2 (9)	
	Protestant or Christian	4 (17)	
	Mormon	3 (13)	
	Muslim	2 (9)	
	None	7 (30)	
	Others	4 (17)	

### Findings

The analysis revealed 5 key categories: reasons for using the app, favorite content, defining spiritual identity, relevance to GenZennials, and recommendations for improvement. Each category encompassed one to several themes (see Multimedia Appendix 1 for themes and accompanying example quotes).

### Reasons for Using the Skylight App

#### Overview

Participants identified multiple reasons for using Skylight including to relax, escape, or ground themselves; improve their mood; and enhance their overall health and wellness. Participants also used the app for its diverse range of content and because it was free to use.

#### Relax or Escape or Ground

Participants used Skylight to divert from stressful situations, center themselves, and relax. Users valued the ability to use the app in times of immediate need.

It just takes the edge off the day...whenever I’m needing to just relax, Skylight has become part of it [the day].

Participant 1004, female

Many users described the app as an escape from daily stressors and current events, providing a much-needed respite from the demands of their lives.

I’m at the tail end of a very nasty breakup from a long term relationship...so there’s been numerous times that’s revolved around that [Skylight use].

Participant 921, female

Just to come back to a center, to a place within me where I don’t feel as bogged down by the world or some of the stresses in life.

Participant 1011, female

In these cases, participants appear to perceive the app as a tool to self-soothe and self-manage immediate and daily stressors.

#### Improve Mood

Some users described instances where they navigated to Skylight to boost their mood when feeling down. This includes feelings of sadness or despair, whether due to a situation that triggered these feelings or because of past experiences.

If I’m feeling...sad...thinking about some of the things I’ve been through can get me a little down. So I’ll open that [Skylight] and it always uplifts me.

Participant 1011, female

#### Overall Health and Wellness

Skylight users described using the app to improve their overall health and wellness.

I’m using the app to help mentally and it helped spiritually as well. I’m just trying to...take it one day at a time...trying to find different methods to help me cope.

Participant 695, female

Some mentioned using specific content, such as affirmations, for their overall health and wellness, including depression and anxiety:

The depression, the self-esteem, the self-worth, you know, there’s so many affirmations online you know.

Participant 1004, female

So I deal with anxiety, and some other things, so being able to, like I’m really big on meditation, and affirmations, and pretty much anything that the app has...and they helped me in my everyday life by creating morning routines.

Participant 1012, female

Many also used Skylight to improve sleep, listening to content before bed or while falling asleep.

So a lot of times I do pick up the sleep and meditate and it actually makes me fall asleep quick.

Participant 695, female

In all of these cases, participants describe using Skylight as a tool for their physical, spiritual, and mental wellness. They are also able to address different facets of their wellness using specific content that they feel is appropriate to their wellness needs.

#### Variety of Content

Users favored Skylight’s diverse range of content, mentioning it as a “one-stop-shop” meeting various needs. The app offers a number of spiritual self-care practices, such as meditation, prayer, yoga, and affirmations. Participants appreciated the ability to choose practices according to their preferences.

How I’m using Skylight...it’s like I’m making my own little cocktail in a way, in any kind of direction of self help.

Participant 1004, female

The ability to make “their own little cocktail” allows users to prioritize their wellness in a personalized manner and continue to adjust what content they choose to consume based on their changing needs.

#### Free

Skylight users were grateful that the app is free, citing this as a key reason they downloaded the app and used it.

The fact that it had no in-app costs, because I’ve gotten into some that once you get into it, the prices are just ridiculous.

Participant 921, female

The cost-free aspect of the app sometimes came as a surprise to users since they expected to have to pay to access more content. However, participants described feeling pleased to find out that all of the app’s content could be accessed at no cost. Another participant regretted not finding the app sooner, as it could have saved on other costs.

I pay for Calm, I pay for Finch, I pay for therapy, I pay for mental health medication. Like that’s a lot. There are so many other bills I already pay...if I had found this app sooner than any of the rest of that I probably wouldn’t have needed [the other apps].

Participant 835, female

### Content Favorites

#### Overview

Though not asked directly, participants organically discussed their favorite parts of Skylight. The main appreciation was for the app’s inclusive content.

#### Inclusive Content

Users favored Skylight’s inclusivity toward diverse faiths, beliefs, and backgrounds. For instance, participants in any stage of their life or spiritual background could participate without feeling excluded or offended. Participants in this study had diverse religious (or nonreligious) upbringings, and regardless of their current beliefs or perspectives on spirituality, Skylight offered a space for them to explore and practice spirituality in a respectful manner.

I’ve got like this spiritual gumbo going on. And I do not feel disrespected in any way.

Participant 894, nonbinary

I like how it doesn’t focus on a specific deity type thing. How it can be applied to any way shape or religion that you are, it can even apply to anywhere. Because mine is kind of fluid. Sometimes it tends to kind of change depending on my circumstances or what I believe.

Participant 289, female

### Defining Spiritual Identity

To understand participants’ spiritual identities, we asked if they considered themselves spiritual and/or religious. The most prominent theme was identifying as solely spiritual. Those identifying as spiritual saw religion as more rigid, such as having established rules and expectations. Spirituality was described as more fluid, allowing the incorporation of various beliefs and practices. One participant described how their past religious affiliation influenced their spiritual identity.

So I’ve come to a space where I want a better, a real relationship with God. But on my terms, rather than on what my parents and my grandparents taught me...But for me, it’s not based on religion because...it was very strict and what you could and could not do, rather than just having a love for God and...taking care of yourself...So I think that’s why I consider myself to be more spiritual because I feel like spirituality comes with something that’s within, not just the external factors, but what’s within you that can kind of bring out the light in those external factors.

Participant 1012, female

For another participant, they reflected on how it was difficult to identify with their family’s religious background given its rigidity, and they had difficulty finding a meaningful connection to this religious belief. As a result, they identified being spiritual as something broader while having a connection to something greater.

...in my family, it was our belief system that was religious based. And that never really seemed to work for me. But as I moved away from it, I still felt a connection to something. I knew something was there...something higher than me. But it didn’t have to be labeled as something...

Participant 1011, female

Both participants reflected on their past religious upbringings and discussed the role they played in their current spiritual identity. They present similarities to how GenZennials as a whole feel about the rigidity of institutionalized religion and how they seek practices that are more flexible and tailored to their beliefs.

### Relevance to GenZennials

#### Overview

When asked about how Skylight was relevant to their generation, 2 themes emerged: content and mental wellness.

#### Content

Participants appreciated Skylight’s content, explaining that it was modern and attractive to their age group. The app’s modern approach appealed to its users because it included practices that are considered newer and more prominent among younger generations.

I think it’s more modern. I don’t know if older generations really...did affirmations and stuff like that...It’s really like...almost making a hip way to practice religion.

Participant 677, female

Users found the succinct, understandable delivery of the content well-suited for GenZennials accustomed to digesting information rapidly. Further, they had a clear understanding and awareness of their technological capabilities and their need for fast, accessible information.

We’re a very fast generation, everything is instant gratification, instant reward...you can have short, less than three minute videos, and that is great for a “high speed, I gotta go now, I gotta move now” generation.

Participant 921, female

#### Mental Wellness

Participants also noted how GenZennials engage with mental wellness (eg, spiritual self-care) differently than older generations. Apps such as Skylight allow GenZennials to cultivate their mental wellness through tailored spiritual self-care practices. This further ties into the previous theme (content), where users appreciated Skylight’s modernity since younger generations participated in spirituality differently than older generations.

I do think that like the people in my generation...they’re generally...a little bit more spiritual and more interested in meditation, breath work, and yoga became so big as I was growing up too.

Participant 1003, female

### Overall Improvement Recommendations

#### Overview

When asked about how to improve Skylight overall, participants primarily suggested increasing personalization features, adding more interactive content and gamified content, expanding health-related offerings (ie, adding more content related to general health), and improving representation across genders and cultures.

#### Personalization

Participants suggested various ways Skylight could make the app more personalized to its users. Suggestions included having notification reminders to log in to Skylight, content bookmarking so they could easily go back to the practices they saved, practice completion tracking so they could track their spiritual practice progress, and customizable playlists to curate their own list of spiritual practices.

I was gonna say if there was a way to make a playlist you know, from everything from the yoga to the frequency music, to you know from every selection they have.

Participant 1004, female

But if you guys do add more content, that [ability to make playlists] might be helpful going forward, just so people can really customize it to their preferences.

Participant 663, female

#### Interactive Content

Participants discussed wanting more interactive content features, such as chat functions (Multimedia Appendix 1) or gamified content. One participant expressed wanting built-in time for reflection during spiritual practices in order to actively engage with the content.

I would want there to be long pauses in between each thought to process what was just said. Speaking from experience, I definitely have found that some follow-alongs that I listened to just say a lot and they’re all really good things. We don’t get time to really process and internalize what they’re saying.

Participant 102, male

#### More or New Content

Participants enjoyed existing content overall, and some wanted to see more variety of content on the app so that they could have more options to choose from.

Definitely more different variety. Different styles. You know, like exercises? Maybe that will be good, not just for your mind but for the physical body.

Participant 821, female

In addition to increasing the variety of current content, participants recommended adding new topics such as mindful eating, recovery support for alcohol and substance use, healing from abusive relationships, and more.

I feel like you should put in a section about eating the right foods for your body since whatever you eat is how you feel. That’s definitely connected to your mind, soul and body.

Participant 817, female

#### Representation

While participants expressed satisfaction with Skylight’s inclusiveness toward different faiths (see the *Inclusive Content* section), they proposed enhancements to improve the representation of GenZennials in the app. For example, one participant proposed adding more male creators since most were female, feeling this could attract and engage more male users.

...if you wanted to appeal to more of a male audience, having more male creators on the app...I think that could help with [my] generation.

Participant 905, male

Another participant recommended increased cultural and language diversity in the app to reach a broader audience. Currently, the app offers music from Korean and Ukrainian artists. Participants saw these inclusions as favorable and expressed wanting more.

Maybe diversify it, like offer other languages or maybe different methods in regards to different cultures.

Participant 992, female

Increasing the representation of different backgrounds in the app may offer another way to increase engagement with the app, as GenZennials see their identities reflected in the app content.

## Discussion

### Principal Findings

The purpose of this paper was to (1) understand why GenZennials use a spiritual self-care app, Skylight; (2) explore how GenZennials identify spiritually; (3) determine Skylight’s relevance to GenZennials; and (4) gather feedback to improve the app. Our qualitative analysis yielded five key categories (each containing one to several themes) including (1) reasons for using the app, (2) favorite content, (3) defining spiritual identity, (4) relevance to GenZennials, and (5) recommendations for improvement. This is one of the first qualitative papers to explore GenZennials’ experience with and perceptions of a spiritual self-care mobile app as well as their spiritual identities. Our findings will inform future spiritual self-care app development and randomized trials.

### Reasons for Using Skylight

Participants reported using Skylight to relax, escape, or ground themselves; improve mood; and enhance overall health and wellness and because the app offers a variety of content and is free. The variety of reasons participants gave for using Skylight reflect the diverse needs of GenZennials. Compared to older generations, GenZennials have been described as “psychologically vulnerable” due to their poorer mental health and increased maladaptive behaviors (eg, substance use and sleep aids) [26]. One-third (3465/10,500, 33%) of Gen Z and one-fourth (2730/10,500, 26%) of millennials report that they use social media to discuss and cope with their health issues rather than seeing a physician [27]. Gen Z are more likely to use digital wellness apps than older generations, as Gen Z are more invested in their wellness [14,28]. Taken together, this generation may benefit from accessible digital tools (ie, apps) that incorporate spiritual self-care to address their diverse wellness needs.

Participants appreciated Skylight’s variety of content (eg, affirmations, prayer, and yoga), allowing them to use the app for multiple purposes, such as relaxation and sleep. Participants mentioned the instances where they used the different content offered through Skylight to address their anxiety, depression, and overall health and wellness. This reflects quantitative findings from a cross-sectional study of 475 GenZennial Skylight users, in which spiritual well-being, overall health, and mental health were the top 3 reasons users downloaded the app [19]. Offering variety in app content may promote continued use, as research shows that lack of content variability contributes to poor adherence in mindfulness apps [29]. Offering content variety may allow users to tap into the many facets of their health, thus supporting their long-term use.

### Content Favorites

Participants appreciated the app’s inclusivity, which made them feel welcomed regardless of their religious backgrounds or beliefs. Other types of digital health apps have been criticized for not providing inclusive content [30,31]. A scoping review of mental health app evaluation frameworks found that only 58% (25/43) of frameworks included a diversity, equity, and inclusion (DEI) criterion; ensuring that apps are inclusive of the individuals they represent is important for user satisfaction, engagement, and retention [31]. GenZennials care deeply about DEI, with 80% (503/603) of Gen Z reporting that it is important for brands or companies to address DEI [32]. Creating inclusive apps is also crucial for app-based interventions to effectively serve diverse communities [31,33].

Participants also discussed using the app because it is free to download and use. Many mental health apps might be free to download, but there are in-app costs (eg, required to pay to unlock the full version). Costs associated with mobile apps can be a barrier to user retention [34,35]. For instance, studies that have explored user behaviors in mobile apps suggest that in-app costs can contribute to negative user perceptions, while free apps are associated with positive user perceptions [36,37]. In a systematic review of 208 papers, app costs were a barrier to using mental health apps [38]. Another study on health app use among 1604 adults (18 to 81 years of age) revealed that cost was a significant concern and a primary reason individuals never downloaded a health app or discontinued using a health app [39]. Over half of GenZennials (ie, 52% of Gen Z and 55% of millennials) report finances as a major factor that negatively impacts their mental health [40]. Notably, most participants in this study (15/23, 65%) had low socioeconomic status, and more than a quarter (6/23, 26%) were students. This demographic is more inclined to use free mental health apps compared to paid alternatives due to financial constraints as full-time students [41]. In sum, digital mental health apps with no costs may improve user engagement and retention in GenZennials.

### Defining Spiritual Identity

In line with previous evidence [9,11,42], most participants reported identifying as solely spiritual when asked if they consider themselves spiritual or religious or both. This study provides further insights into the spiritual identities and practices of GenZennials. Those who were identified as spiritual explained that previous experiences with organized religion led them to adopt a more spiritual identity.

They embraced the fluidity of spirituality as it contradicted the rigidity of religion that they had experienced from their past religious affiliations or from being raised in a religious household. Participants also discussed how spirituality, as opposed to religion, allows them to incorporate different beliefs and practices, which is consistent with the concept of “faith unbundled” [10]. In a study of 475 GenZennials, over one-third (n=142, 36%) defined spiritual self-care as practices that support their connection to something greater than themselves (ie, higher power, nature, and community) [19]. Overall, participant reflections are consistent with GenZennials’ tendency to disengage with organized religion and preference to autonomously practice spirituality [43].

### Skylight Relevance to GenZennials

Participants reported that Skylight’s content was relevant to their generation because the content was modern. Participants explained that the app was modern because it is a more “hip” (eg, affirmations) way to have religious and spiritual practices. Compared to older generations, GenZennials are more likely to either abandon the religion they were raised in or never identify with a religion at all [43]. However, GenZennials draw from a variety of religious and spiritual backgrounds to craft their own spirituality or religion with many who engage in more modern practices [44]. In a survey with over 10,000 Gen Z, 40% (4110/10,274) were more likely to practice yoga as a spiritual practice than attend a religious service (2569/10,274, 25%) and were more likely to go in nature (4623/10,274, 45%) or meditate (1006/10,274, 39%) than read religious text as their spiritual practice [10]. This study suggests that a spiritual self-care app with a contemporary approach to spirituality may resonate with GenZennials due to the alignment with their inclination toward modernized spiritual practices.

Participants shared how Skylight’s content was easy to integrate into their daily routines, particularly because the content was short (<3 minutes). Mental health app users prefer apps that support their routines and are more likely to continue using apps that are easier to use [45] and not time-consuming [46]. While research suggests mindfulness practices (eg, meditation) may require 10 to 30 minutes to positively impact mental well-being [47,48], user retention is another component that should not be overlooked. Recent research suggests that short but consistent engagement with mobile health apps is associated with improved health behaviors, emphasizing the significance of smaller doses for engagement and improved outcomes [49]. Increasingly popular practices today include single-session interventions for mental health [50], 5-minute breath work [51], and brief meditations [52]. GenZennials want quick content, and results from this study underscore the importance of delivering brief content in spiritual self-care apps for GenZennial users. Future research should explore the effects of smaller doses of spiritual self-care practices and their impact on spiritual and mental well-being, especially in GenZennials.

### Recommendations for Improvement

Participants in this study desired more personalization capabilities in the Skylight app, such as creating their own playlists or having a habit-tracking feature. GenZennial consumers want to see personalization in products [53]. In an evaluation of 104 mobile mental health apps, which included an analysis of 88,125 user reviews, personalized features (eg, the ability to log activities and set goals) were a primary feature app users favored [45]. Digital mental health apps with personalization features have demonstrated better adherence than those that do not have personalization capabilities [54]. A systematic review of digital mental health interventions concluded that machine learning (ie, algorithm-based tailoring) should be leveraged for more personalization of digital mental health services to improve outcomes [54]. In summary, spiritual self-care apps should include features that allow users to personalize their experience.

Participants discussed wanting the app to be more interactive. Some users desired the ability to chat with others, either through a dedicated chat function or by using a comment section on content, while others desired more interactive content where they could actively engage (ie, pauses for reflection during guided content). These findings align with previous research, where investigators found that app users enjoyed digital mental health interventions with interactive and gamified content in a qualitative synthesis of 41 studies [55]. Interactive apps are not only desired and favored by users, but they also demonstrate better effectiveness in improving mental health than those that are not interactive. A systematic review of 15 studies found that digital mental health interventions with interactive content and features (ie, multimedia lessons, web-based exercises, and game-based challenges) were more effective in reducing depression symptoms than interventions with no interactive components [55]. Notably, these results in the review were found among interventions with no human interaction components (ie, live chat with professionals), which are usually resource-intensive (eg, costs) [55]. Incorporating interactive components into apps may be a cost-effective way to engage and retain users through improving the user experience.

While participants in this study enjoyed the existing content on Skylight, they desired new and more content moving forward. Digital mental health apps must consistently update their content to retain users [45]. In an analysis of 104 mental health apps, users felt bored and limited if there was not enough content in the app [45]. However, in a study analyzing 5 million user reviews of mobile health apps, users were frustrated when apps had more content than needed [56]. Future research is needed to explore how much content is enough to allow users to manage their mental health and well-being while minimizing overwhelm. Participants in this study suggested adding new topics to Skylight content. Suggested topics included, but were not limited to, managing interpersonal relationships, recovery from drug and alcohol use, and parenting. This is unsurprising considering that these are related to the common areas of concern for GenZennials [57]. An analysis of 41 qualitative studies showed that users using a mental health app preferred and liked when the app content was relatable to situations they were going through [55]. Developers of mental health apps specific to GenZennials should consider delivering content that is relatable to GenZennials’ daily tasks, relationships, and situations.

When discussing how Skylight was relevant to their generation, GenZennials in this study made recommendations for improved representation. Participants suggested adding more male creators and more representation of different cultures and languages. Male individuals are less likely to use well-being apps compared to female individuals [58]. However, the reasons behind this lower use remain uncertain—it could stem from general disinterest among males in wellness apps, insufficient male representation in app content and marketing, or a lack of customization catering to male individuals’ needs. On the contrary, in a recent cross-sectional survey that explored perceptions of the Skylight app and self-reported mental health, almost half of the participants were male [19]. Qualitative health research has a persistent gender bias, with a higher participation rate of female individuals compared to male individuals, partially due to challenges in recruiting male individuals for health studies [59].

Participants in this study also recommended offering more languages and incorporating more cultures in the app. GenZennials are more racially or ethnically diverse than older generations [60]. Spiritual self-care mobile apps need to be designed with and informed by individuals from a variety of cultural and racial or ethnic backgrounds. These are concerns that are not exclusive to spiritual self-care apps. In fact, digital health apps as a whole lack effectiveness in reaching those from varied cultural backgrounds [33,61]. Current mobile mental health app evaluation frameworks lack consideration of DEI criterion in their framework [31]. There is a significant need for improvement in the development and design of digital mental health apps to ensure they accurately represent and cater to target populations.

### Limitations

This study is not without limitations. First, eligibility criteria did not specify a minimum app use duration, and we did not gather data on use time. This may have limited insights, as participants likely varied in their frequency and extent of app use. Second, our sample was majority female and White, which limits the generalizability of our findings. Female individuals are more likely than male individuals to use well-being apps [58], and this was reflected in the sample of our study. Female individuals who use Skylight may have different motivations for using Skylight than male individuals and engage with the content differently. However, this does not indicate that male individuals are not using the app [19]. In addition, the sample was majority White, which limits our understanding on how individuals from other racial and ethnic backgrounds engage with Skylight’s content and perceive the inclusivity of the app. Nonetheless, our findings still shed light on the perspectives and experiences of GenZennial users of a spiritual self-care app, particularly among those from low socioeconomic backgrounds as more than two-thirds (15/23, 65%) of our sample had an annual income less than US $50,000. Third, qualitative research is subject to biases in analyzing and interpreting data. However, data were analyzed by 2 researchers who practiced reflexivity (ie, discussed potential biases throughout the study and analyses).

### Future Research

Our findings shed light on future research necessary (Textbox 1) to fill knowledge gaps related to improving spiritual and mental well-being in GenZennials using a mobile app.

**Summary of key findings and future directions for research.**

**GenZennials use a spiritual self-care app for various health and wellness needs**

- Explore the effects of using a spiritual self-care app for multiple wellness needs on GenZennial mental health outcomes.
- Determine the effect of content relevance to GenZennial problems (ie, substance use) on retention or engagement.

**GenZennials appreciate inclusive content in a spiritual self-care app**

- Determine the effects of inclusive app content on user engagement across GenZennial demographics (eg, racial or ethnic diversity of app users).

**GenZennials primarily use a spiritual self-care app because it is free**

- Examine the effects of offering spiritual self-care app free of cost on user engagement and retention in GenZennials.

**GenZennials mainly identify as solely spiritual**

- Determine the acceptability and usability of a spiritual self-care app among GenZennials who identify as religious.

**GenZennials explained the spiritual self-care app was relevant to their generation because the content was short**

- Explore the effects of smaller doses of spiritual self-care practices and their impact on spiritual and mental well-being in GenZennials.

**GenZennials suggest increasing male representation in the app, and few males participated in this study**

- Recruit more GenZennial male individuals to gather their insights and feedback on spiritual self-care apps.
- Explore quantitative data to confirm usage patterns among male individuals since male individuals are difficult to recruit and may not reflect the number of male app users [58].

**GenZennials suggest adding more interactive features to a spiritual self-care app**

- Examine the effects of interactive components (eg, anonymous chat groups, playlists with favorite content, and pauses for reflection) in a spiritual self-care app on GenZennial spiritual and mental well-being outcomes.

### Conclusions

This is the first study to explore how GenZennials view a spiritual self-care app for enhancing their spiritual and mental well-being, offering important insights into GenZennials’ spiritual identities and the relevance of these apps to their generation. This study also provides GenZennials’ recommendations for improving a spiritual self-care app design and features. These findings may also guide future mental health app developers seeking to create spiritual products and content tailored to GenZennials. Interviews with GenZennial Skylight app users revealed that they used the app for various reasons related to their overall health and wellness. Users appreciated the variety of content in the app, and several users cited the app’s completely free access as their primary reason for using it. Considering most participants were from low socioeconomic backgrounds, our findings underscore the value of providing a free and accessible spiritual self-care app for GenZennials. The inclusivity of the app was another notable feature users mentioned when describing what they enjoyed about the Skylight app. Consistent with the GenZennial population, most participants identified as solely spiritual. Our findings should inform the future creation and improvement of spiritual self-care apps aimed at cultivating GenZennials’ spiritual and mental well-being.

## Appendix

Multimedia Appendix 1 Themes and example quotes.

## Abbreviations

DEI: diversity, equity, and inclusion
Gen Z: Generation Z
