# Supplementary material for: Exploring Generation Z and Young Millennials’ Perspectives of a Spiritual Self-Care App and Their Spiritual Identity (Skylight): Qualitative Semistructured Interview Study
Source: JMIR Form Res. 2023 Dec 28;7:e54284. doi: 10.2196/54284 (PMC10784987; doi:10.2196/54284)
Supplement: Multimedia Appendix 1 [file formative_v7i1e54284_app1.docx]

**Multimedia Appendix 1.** Themes and example quotes.

| **Categories** | **Themes** | **Example Quotes** |  |
| --- | --- | --- | --- |
| App Use Reason | Relax/Escape/Ground | “I feel like my brain just kind of goes on autopilot and I’m dissociating a lot so I feel like I need something…like Skylight to ground me.” (Participant 817, female)  “When I get like tense moments of parenting…I’m just like having mommy meltdowns..I just need a moment, like 10 minutes to just kind of regather myself. And I found with the app it’s just so beneficial…it’s [Skylight app] really self soothing. You learn to like self soothe yourself in different ways.” (Participant 992, female) |  |
|  | Improve Mood | “I can be like down about something or frustrated with what I’m doing, no matter what it is. I just turn on Skylight to feel alright.” (Participant 821)  “I noticed four key points that keep bother me in life like mentally, spiritually, financially and physically so it’s like the app is helping me…I could just sit here on the app and just…go through everything and it helps me.” (Participant 695) |  |
|  | Overall Health and Wellness | “I noticed four key points that keep bothering me in life like mentally, spiritually, financially and physically so it’s like the app is helping me…I could just sit here on the app and just…go through everything and it helps me.” (Participant 695, female) |  |
|  | Variety of Content | “What you need is there if you want to look for it. Also, like you can just focus on one thing. This is the app I use for movement…for meditation.” (Participant 835, female) |  |
|  | Free | “I think I [would like to use Skylight app] more especially because it’s free.” (Participant 905, male)  “The part I like best about it is you don’t have to pay for it. Because that’s the biggest issue with all those kinds of apps. And if it’s like free help that I’m getting, and it's informative, and it’s gonna help me then that’s like the reason I use it.” (Participant 817, female) |  |
| Content Favorites | Content: Inclusive | “I think for me… what makes Skylight unique is that, you know, I think a lot of people are aware of Calm and InsideTracker, and all these different apps, but nothing that is centered around spirituality… there is that appetite to have something that is centered around spirituality but still inclusive. And so for me the distinguishing factor is that [Skylight] is…centered around spirituality.” (Participant 832, female) |  |
| Defining Spiritual Identity | Solely Spiritual | “I'm not so much [religious] because of my upbringing, because in my family, it was our belief system that was religious based. And that never really seemed to work for me. But as I moved away from it, I still felt a connection to something. I knew something was there, something higher, something higher than me…There's something out there looking out for me. There's just too much beauty in the world.” (Participant 1011)  “I would consider myself spiritual and non religious.”  (Participant 992) |  |
| Relevance to GenZennials | Content | “I think it’s really relevant to my generation because...it allows people my age who might be busy with class to use it under five minutes. Like they’re very helpful videos…that are only three minutes long…This generation is looking for apps like this. Easy interface, easy to use, that allow for quick exercises.” (Participant 905, male) |  |
|  | Mental Wellness | “I think the app is absolutely aimed towards our generation because older people are still happy…I think it’s definitely aimed towards our generation so that we can help the younger generation…be better hopefully.” (Participant 1006, female) |  |
| Overall Improvement Recommendations | Personalization | “I was gonna say if there was a way to make a playlist you know, from everything from the yoga to the frequency music, to you know from every selection they have.” (Participant 1004, female)  “A habit tracking view, being able to see what your history is, what you've been able to complete. That could be nice, especially if you're more habit oriented. And you'd like to see that progress of completing certain exercises every single day.” (Participant 905, male) |  |
|  | Content: Interactive | “Maybe a chat feature…where people can talk to each other…you never know who's going through what… even if it's just a comment section, where people are just chiming in…you know, encouraging one another…something like that” (Participant 695, female) |  |
|  | Content: More/New | “I would honestly probably just add more…of just everything…I would say just more content, more content.” (Participant 874, female)  “A lot of people are probably in recovery, looking for things just to keep them uplifting, and to keep them going because it's such a hard thing. Especially at first, it's like those first few months, like the hardest. I think if you'll have a section maybe for people that are experiencing hardship with a directive affirmation set. I think that will be beneficial.” (Participant 466, female) |  |
|  |  |  |  |
|  | Representation | “You know, hopefully they [Skylight] do take up the opportunity that's given on the website, on the app…have the language variety...because there are so many people in my age that I know that don't speak anything [but] English…or [just] Spanish. (Participant 1004, female) |  |
